# Using a Machine Learning Approach to Predict Outcomes after Radiosurgery for Cerebral Arteriovenous Malformations

**DOI:** 10.1038/srep21161

**Published:** 2016-02-09

**Authors:** Eric Karl Oermann, Alex Rubinsteyn, Dale Ding, Justin Mascitelli, Robert M. Starke, Joshua B. Bederson, Hideyuki Kano, L. Dade Lunsford, Jason P. Sheehan, Jeffrey Hammerbacher, Douglas Kondziolka

**Affiliations:** 1 Department of Neurosurgery, Icahn School of Medicine at Mount Sinai, New York City, NY, USA; 2 Department of Genetics and Genomic Sciences, Icahn School of Medicine at Mount Sinai, New York City, NY, USA; 3 Department of Neurosurgery, University of Virginia Medical Center, Charlottesville, VA, USA; 4 Department of Neurosurgery, University of Pittsburgh Medical Center, Pittsburgh, PA, USA; 5 Department of Neurosurgery, New York University Langone Medical Center, New York City, NY, USA

## Abstract

Predictions of patient outcomes after a given therapy are fundamental to medical practice. We employ a machine learning approach towards predicting the outcomes after stereotactic radiosurgery for cerebral arteriovenous malformations (AVMs). Using three prospective databases, a machine learning approach of feature engineering and model optimization was implemented to create the most accurate predictor of AVM outcomes. Existing prognostic systems were scored for purposes of comparison. The final predictor was secondarily validated on an independent site’s dataset not utilized for initial construction. Out of 1,810 patients, 1,674 to 1,291 patients depending upon time threshold, with 23 features were included for analysis and divided into training and validation sets. The best predictor had an average area under the curve (AUC) of 0.71 compared to existing clinical systems of 0.63 across all time points. On the heldout dataset, the predictor had an accuracy of around 0.74 at across all time thresholds with a specificity and sensitivity of 62% and 85% respectively. This machine learning approach was able to provide the best possible predictions of AVM radiosurgery outcomes of any method to date, identify a novel radiobiological feature (3D surface dose), and demonstrate a paradigm for further development of prognostic tools in medical care.

Predicting the outcome of a specific patient treated with a particular therapy is fundamental to medical practice. In the case of cerebral arteriovenous malformations (AVMs), several scoring systems have been developed to augment clinician experience in predicting individual patient outcomes after treatment with radiosurgery, a type of highly focused radiation therapy^1^,^2^,^3^,^4^,^5^,^6^,^7^,^8^. These outcomes, including the probability of treatment success, the expected morbidity associated with a given therapy, and the expected latency period between treatment and obliteration, all factor into the clinical decision to treat AVMs with surgery, embolization, radiosurgery, or a multimodal approach^7^,^8^,^9^,^10^,^11^,^12^,^13^,^14^,^15^,^16^. The degree to which we can improve upon currently available classification systems for predicting AVM treatment outcomes is unknown.

Machine learning is an interdisciplinary field combining computer science and mathematics to develop models with the intent of delivering maximal predictive accuracy^17^. Combining these new analytical tools with modern clinical databases and registries promises an entirely new approach towards conducting medical research and, ideally, developing ways to predict individual outcomes and the risk to benefit profiles from specific therapies^18^. In the present study, our aims are to (1) apply a machine learning approach towards predicting individual patient outcomes after AVM radiosurgery and (2) analyze the predictive capability of existing grading systems for AVM patients treated with radiosurgery.

## Results

### Data and analysis pipeline

**Figure 1:**
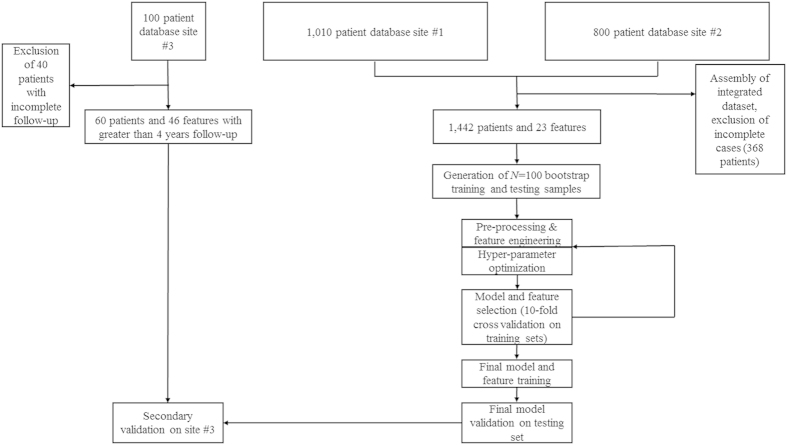
Flow chart of data assembly, processing, and analysis. Patients were gathered from two independently maintained, prospective AVM radiosurgery databases. Both databases were integrated into a single dataset of 1,810 patients described by 23 features. The features were divided into an arbitrary number of bootstrap training and testing samples (N = 100). After data pre-processing including standardization and normalization, an iterative process of feature engineering, feature selection, predictor generation and assessment was instituted. After a satisfactory set of features was selected, a hyperparameter optimization routine was utilized, and four final predictors were trained and cross-validated on the dataset. The predictors included a logistic regression model, a random forests model, a stochastic gradient descent model, and a support vector machine model. Existing clinical models of AVM outcomes were also tested on the dataset, including the Spetzler-Martin scale (SM), modified Radiosurgery Based AVM Score (RBAS), and Virginia Radiosurgery AVM Scale (VRAS).

Of the 1,810 patients, a varying number were excluded for incomplete baseline data or failure to achieve the endpoint by a given time threshold leaving 1,291–1,674 patients at each time threshold with complete 23 feature profiles which were graded utilizing current prognostic models for AVM radiosurgery outcomes (RBAS, VRAS, SM) (Fig. 1, Table 1). Of the patients considered as having been obliterated, 76.8% were confirmed with cerebral angiography, while 23% were noted on magnetic resonance imaging (MRI) only. The 1,442 patients were randomly sorted 2:1 into 100 training and testing sets of 961 and 481 patients, respectively (Table 2).

### Feature selection

After pre-processing and feature engineering, the automatically generated features failed to yield any appreciable benefit and were subsequently dropped, leaving 23 features for use in classifier construction (Table 3). The top three features in the LR predictor at every time point were maximum diameter (frequency = 1), prior embolization (frequency range* = 0.88–1*), and margin dose (frequency range = 0.92–0.97). The top five features at eight years were equivalent to the top five features at six years: (1) maximum diameter, (2) prior embolization, (3) margin dose, (4) number of isocenters, and (5) brainstem location. These were also, on average, the most useful features across all time points. For predicting obliteration at two years, a notably different set of features was most useful: (1) maximum diameter, (2) prior embolization, (3) margin dose, (4) 3D surface dose, and (5) isodose. 3D surface dose did not contribute significantly to predictor performance, with the exception of margin dose at the two-year time point. Various locations were always present in a large number of models, with more important locations including brainstem (frequency range = 0.63–0.96), thalamus (frequency range = 0.75–0.88), and temporal lobe (frequency range = 0.68–0.91).

### Predictor accuracy at predicting favorable outcomes

**Figure 2:**
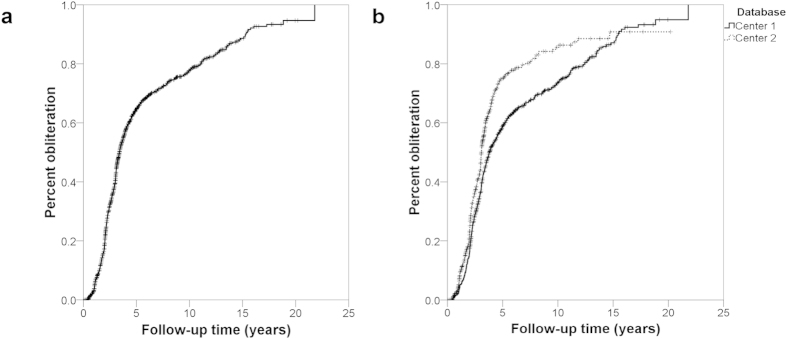
(**a**) Kaplan-Meier plot of obliteration over time of the combined dataset, showing a median time to obliteration of 3.4 years (95% C.I. 3.2–3.6 years), and an average time to obliteration of 6.1 years (95% C.I. 5.8–6.5 years). (**b**) There was a divergence in results between treatment sites, with one site reporting an average time to obliteration of 6.7 years (95% C.I. 6.2–7.1 years), and the other reporting an average time to obliteration of 5.2 years (95% C.I. 4.6–5.8 years) (p < 0.001).

**Figure 3:**
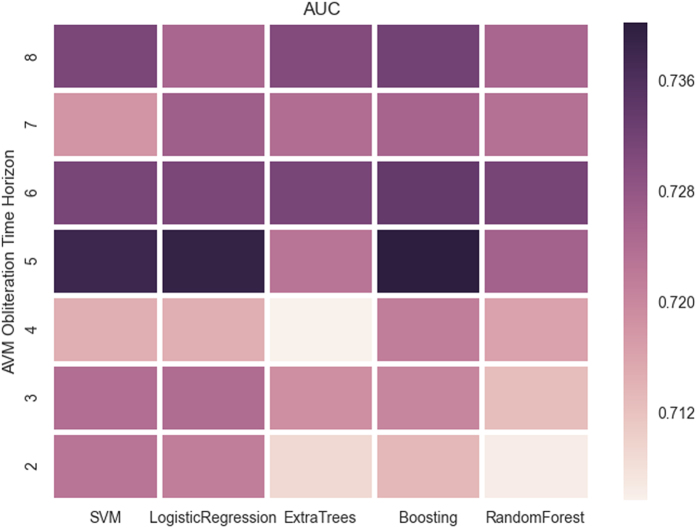
A heatmap of AUC for the different classifiers across various times points. There is a trend towards peak performance at five years for all classifiers, with a skew towards superior performance at later time points. The increased accuracy at later time points is likely due to the more evenly balanced data due to more patients having met the endpoint by later time points.

**Figure 4:**
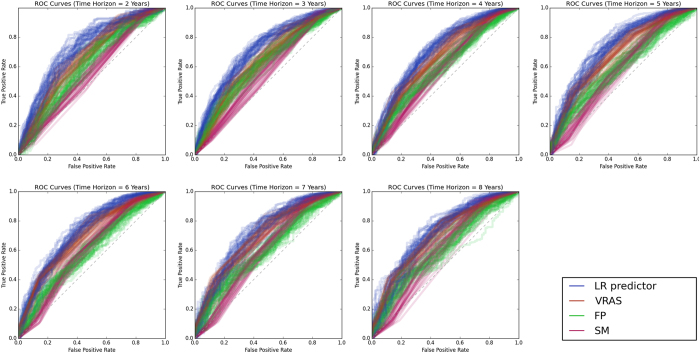
ROC curves for four predictive models of AVM outcomes after radiosurgery at all assessed follow-up time points on the testing set. There is a noticeable decay in accuracy for all models at longer follow-up time points. The LR predictor consistently delivers more accurate predictions of individual “favorable” outcomes than existing systems.

**Figure 5:**
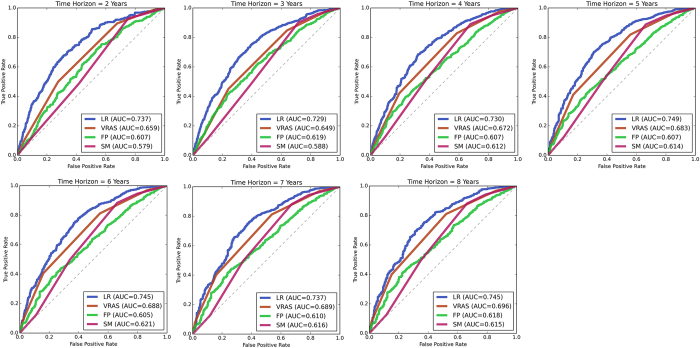
ROC curves for the trained classifiers were tested on a heldout dataset from Site #3 demonstrating similar results and potential for generalization to other institutions and clinical settings.

**Figure 6:**
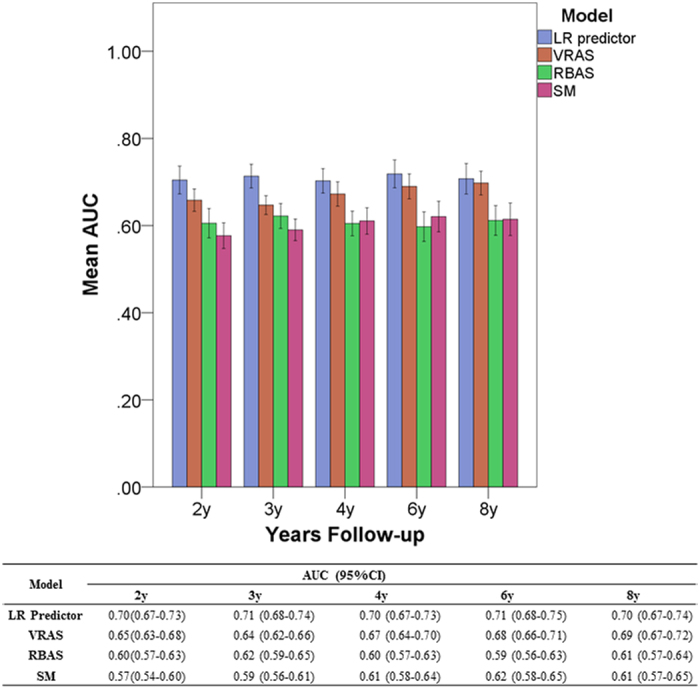
Comparison of AUC with 95% CIs for the tested models at various time points. While the models have a consistent hierarchy of accuracy at most time points with the LR model being the most accurate and the SM model being the least, there is a trend of increasing predictive accuracy with time for the VRAS model. This potentially suggests capture of more time-dependent features in the VRAS model compared to the other models, which have relatively constant accuracy across all time points.

The median time to a favorable outcome was 4.2 years (95% CI 3.8–4.7 years; Fig. 2). Predictors were optimized and subsequently tested on the AVM dataset utilizing the previously mentioned features. Without hyperparameter optimization, LR provided superior classification results in numerous trials. However, with optimization, all classifiers were able to obtain similarly good results (Fig. 3). At all follow-up time points, the LR predictor had superior AUC compared to the VRAS, SM grade, and RBAS (Fig. 4). The LR predictor additionally continued to outperform existing models on the heldout dataset from Site #3 (Fig. 5). The LR predictor delivered superior predictive accuracy compared to existing clinical models with an AUC of 0.70 (95% CI 0.67–0.73) at four years, which was relatively constant at all times points (Fig. 6). The VRAS had a steady gain in accuracy over time, with an AUC at two, three, four, six, and eight years of 0.65, 0.64, 0.67, 0.68, and 0.69, respectively. Using four years as an example time point, the LR predictor had an AUC of 0.70 (95% CI 0.67–0.73), the VRAS of 0.67 (95% CI 0.64–0.70), RBAS of 0.60 (95% CI 0.57–0.63), and SM of 0.61 (95% CI 0.58–0.64).

Compared to alternative linear, ensemble, and support vector techniques, the LR predictor obtained similar results (Fig. 3). At six years, LR had an AUC of 0.72 (95% C.I. 0.68–075), compared to RF at 0.70 (95% C.I. 0.67–0.73), and SVM at 0.62 (95% C.I. 0.56–0.68). The relative performance of all predictors was similar across all time points, despite the difference in predictive algorithms and feature utilization between each technique. Site #3 had 60 patients with at least four years of clinical follow-up. When ML predictors were tested at four years, the best performing aws the LR predictor with an AUC of 0.79 (95% C.I. 0.76–0.81). The LR predictor at four years of follow-up had a sensitivity of 85%, and a specificity of 62% resulting in a positive predictive value (PPV) of 74% and a negative predictive value (NPV) of 76% (Table 4).

### Clinical observations

For patients predicted to have an unfavorable outcome at eight years, there was a trend towards a higher rate of post-radiosurgery hemorrhage (13.5%) compared the favorable outcome group (6.3%, p = 0.089), with a difference in average at-risk years for hemorrhage of 4.9 years in the unfavorable outcome group vs. 4.1 years in the favorable outcome group. Notably, this unfavorable outcome group had no significant group differences compared to the patients having a neutral or favorable outcome.

In order to uncover the importance of 3D surface dose to the predictors, we retroactively analyzed its association with other patient features and outcomes. At two years, 3D surface dose was associated with adverse events. A greater average 3D surface dose was delivered to patients who experienced a post-radiosurgery hemorrhage (252 vs 169 Gy*mm^2^, p < 0.001) and to patients who suffered a permanent neurological deficit (222 vs 196 Gy*mm^2^, p < 0.001). A 3D surface dose greater than 199 Gy*mm^2^ was delivered to 70% of patients who had experienced a post-radiosurgery hemorrhage (p < 0.001) at two years of follow-up.

## Discussion

This study demonstrates a machine learning approach towards medical prognostication, specifically, a novel method for predicting AVM radiosurgery outcomes. While the final predictor from this study has clinical significance in its own right as the most accurate existent predictor of outcomes after AVM radiosurgery, the general approach employed for constructing it is far more important. The application of machine learning methods to medical data constitutes the analytical backbone of a novel research paradigm of: (1) generating large clinical registries or datasets followed by (2) pre-processing and feature selection in order to (3) select and applying machine learning techniques to (4) generate clinical insights and, ideally, (5) actionable principles that may be implemented in clinical practice. In this study, we have succeeded in demonstrating this approach towards medical research utilizing a multicenter cohort of patients harboring cerebral AVMs who underwent treatment with radiosurgery. We also described the final predictor, which currently stands as an accurate and rigorously vetted predictor of individual patient outcomes after AVM radiosurgery.

Given the young age at which AVMs are diagnosed and the devastating neurological consequences of rupture, there is significant interest in predicting AVM outcomes after a given therapy^2^,^19^,^20^,^21^. The SM grading system is the most commonly used classification scheme to predict surgical morbidity based on AVM size, critical function, and venous drainage pattern^22^. Subsequently, new systems were developed to specifically predict AVM radiosurgical outcomes, including the RBAS, which takes into account patient age, lesion volume, and lesion location^4^,^6^,^23^. More recently, the VRAS utilized AVM volume, eloquent location, and prior hemorrhage to predict favorable outcome, defined as obliteration without post-radiosurgery hemorrhage or permanently symptomatic radiation-induced changes, after radiosurgery^24^. All of these systems share a common approach towards construction by starting with clinician-specified variables, and then utilizing the coefficients from subsequent regression modelling to construct an intuitive model that is validated against the original database from which they were derived. The Spetzler-Martin grade remains useful for the classification of AVMs undergoing treatment with radiosurgery^11^,^25^,^26^,^27^,^28^. Interestingly, the VRAS scale had a relatively good performance across several time points, suggesting that it might better capture time independent factors than the other systems. Efforts are currently under way to validate the RBAS and VRAS in a multicenter cohort.

The novel predictors described by the present study, particularly our LR predictor, have a radically different design process. Starting with a large, multicenter cohort dataset assembled from two prospective collected databases, we utilized all available features within the dataset as our initial input, rather than restricting ourselves to clinician-selected features. To further maximize the extent to which we could leverage the data, we then performed several iterations of feature engineering. After screening all engineered features, we discovered that *AVM surface area* multiplied by *margin dose* was particularly helpful for fitting the predictors at early time points. We termed this parameter 3D surface dose, since it effectively describes the total dose delivered to the entire surface/margin of the lesion, and it may have clinical significance in its own right (as discussed below) beyond simply increasing predictive accuracy at early time points. We then turned to identifying optimal predictors in order to achieve the highest possible predictive accuracy while utilizing cross-validation to decrease the risk of over fitting the data. To further ensure accuracy and generalizability, we validated the final models on patients from a third site, which was entirely excluded from model construction. This general process of model training and testing on a multicenter dataset, with validation on independent datasets from other sites is a crucial best practice for prognostic model creation which is often lacking in the construction of existing clinical scoring systems^29^.

This approach has several additional theoretical advantages beyond its increased accuracy and data inclusive design. First, as a computer-based predictive model that is scripted to run off of an arbitrary database, the present model has the advantage of being able to be seamlessly updated as new information becomes available, thus allowing it to adapt with time to shifts in practice patterns and patient populations. This also parlays into an advantage of being distinctly translatable into a practice-specific tool. Rather than being a static construction off of a single center’s experience, to be generalized with unknown error to specific practices and specific patients, the present approach can be run off of any supplied database. The end result is maximally accurate results for clinical decision support in the management of individual patients rather than broadly generalizable knowledge about a disease state. Lastly, by utilizing machine learning methods rather than a developed scoring system, the present approach can be easily modified to account for missing data. The 368 patients which were excluded from the present study were excluded because the scoring systems (VRAS, SM, etc…) could not be calculated for them. It would have been a straightforward process to incorporate all 368 of them into the machine learning models however.

For some of the classical models, results at certain time points are scarcely better than chance. This possibly stems from their limited feature space, with many only utilizing two or three features, or their susceptibility to error stemming from limited sources of data and potential bias prior to analysis. The present study’s predictors are originally designed to mitigate some of these sources of error, as well as benefitting from a larger, more diverse multicenter dataset. Despite these advantages and the reasonable sample size, our predictors also appeared to reach a upper bound on their predictive accuracy around 70%, despite the use of both straightforward and more sophisticated methods. Also, notably, the logistic regression classifier with its sparse, linear feature space performed exceptionally well. We considered utilizing a naïve Bayes classifier as well due to their efficiency and accuracy at classification. However, the conditional independence assumption is clearly *not* true for clinical datasets containing both pre-treatment and treatment features since it is characteristic of treatment features to be dependent upon pre-treatment features.

Several possibilities exist for the cause behind this upper bound on our predictive capabilities and the failure of nonlinear classifiers to outperform simple linear ones. We speculate that intrinsic characteristics of the AVMs including angioarchitecture (how the blood vessels are arranged), hemodynamics, and radiobiology play a large and unknown role in determining patient specific response to radiosurgery^30^,^31^. A recent study by our group noted a potential role for angioarchitecture in determining obliteration rates^9^. Unfortunately, the present dataset did not contain comprehensive information on angioarchitecture for us to include in our analysis. Hemodyanmic features like AVM blood flow, volume, and pressures, may also be crucial to determining the response to radiation, as well as features describing the radiation dosimetry may be helpful for increasing our predictive accuracy. The sample size itself also presents an interesting conundrum. From a statistical perspective it is somewhat small, only consisting of a few thousand patients. For this given pathology, however, the size of the analyzed dataset is enormous, constituting, to the author’s knowledge, the largest AVM analysis published to date. Perhaps most significantly for the case of the more advanced learning models, a significant source of error incurred while learning a feature space may stem from an iatrogenic loss of interesting clinical variants/cases.

Clinicians practically limit the values of treatment features based on clinical features in order to achieve an intended clinical outcome. While this is the essence of medical practice, it does pose a barrier to machine learning by denying predictive models the knowledge of how outcomes are dependent upon those specific interactions which clinicians are avoiding. For example, in the present analysis, brainstem and insular AVMs all have relatively conservative dose regimens. While this approach may yield favorable clinical outcomes, it simultaneously denies a learning algorithm the ability to know that a high dose delivered to the brainstem is associated with adverse events. We suspect that this is partially why more advanced techniques like random forests do not outperform linear models like logistic regression, and why location isn’t as important a feature as one would expect (because doctors are eliminating instructive cases as to its importance). A possible solution to this issue may be utilizing artificial training sets to pre-train models with existing medical knowledge prior to learning from actual clinical cases. This clinical pre-training step would be somewhat analogous to the unsupervised pre-training of convolutional networks prior to supervised learning. We suspect further investigations in this particular problem area will prove essential and fruitful towards increasing predictive power.

An essential component of a machine learning analysis is the generation of actionable insights consequent to constructed predictive models. The LR predictive model is, itself, a valuable clinical tool. As previously discussed, both its superior predictive capabilities as well as its natural ability to be tailored to a particular population or practice make it ideal for implementation in clinical practice. To further demonstrate the practical utility of this approach, we conducted a secondary analysis to evaluate patients who were predicted to have an unfavorable outcome, as well as a secondary analysis of our engineered feature, 3D surface dose.

We looked at the subset of patients who were predicted to have favorable outcomes at eight years by the LR model. This set of patients had a longer time to obliteration, although it was not significant, and despite the difficulty of categorizing patients eight years out from a treatment, the predictor yielded mediocre results at identifying unfavorable outcomes. However, on our subset analysis of the unfavorable outcomes that were identified, we noted an increased rate of post-GK hemorrhages, perhaps due to their longer at-risk period. It is worth noting that, methodologically, this separate analysis could have been avoided, and possibly even improved upon, by constructing an entirely separate classifier designed to exclusively isolate individuals at risk for hemorrhage, rather than attempting to parcel out and identify an at-risk subset from the predictions of favorable outcomes.

The engineered feature, 3D surface dose, was of particular interest due to its utility at early time points, time dependence, and relatively intuitive clinical interpretation. Under the assumption that its significance to the model might be to indicate early events, we looked for correlations between 3D surface dose and clinical events. While loosely associated with obliteration rates, 3D surface dose was significantly associated with adverse events within the first two years. After engineering a new categorical feature, we identified a cutoff of 199 Gy*mm^2^, above which patients at all times points had a greater incidence of treatment-related adverse events. While needing independent verification, a potential mechanism is that 3D surface dose is associated with both increased target edema leading to swelling, deficits, and potentially hemorrhage secondary to shift. A second potential mechanism is that 3D surface dose correlates closely with the dose delivered to surrounding normal tissues. Both of these mechanisms have been observed in other cases. In the case of intracranial radiosurgery for meningioma, surface area and dose are associated with increased intracranial edema^32^,^33^. Modelling studies have also identified surface area alone as correlating with intracranial V-12 (the volume receiving 12 Gy), which is associated with normal tissue toxicity, as well as being predictive of rectal toxicity after prostate SRS^34^,^35^,^36^.

Although the data in this study was collected in a prospective fashion, validated against an independent third dataset, and the study’s analysis was conducted utilizing techniques to minimize bias, it is a retrospective project that requires validation in a prospective manner to demonstrate predictive capability. Regardless of dataset size, quality, and the use of techniques such as cross-validation, there is no internal panacea for validating predictive models which ultimately need external validation^37^. This problem was demonstrated clearly in the present study when attempts to fit models on one center’s data and then test on the other center were challenged by significant variation within each center’s treatment patterns. Only by combining centers, at a slight loss of accuracy, could we develop a more generalized result. Therefore, validation using patient cohorts from other institutions is essential to improving upon these results. The quality of any predictor is ultimately limited by the quality of its data as well, and as noted previously, there are intrinsic limitations to any given dataset – some of which are iatrogenic and clinically desirable, although disadvantageous for modelling. Larger datasets with more variation will prove essential towards increasing predictive accuracy.

Despite the success of this project at generating superior predictors, the results are nonetheless less than ideal for use in clinical practice to guide individual patient management. A potential limitation, with regards to accuracy, is the non-exhaustive search through existing machine learning models. While we utilized a wide range of classifiers, there are many more which we have not yet attempted and may yet yield superior results. As noted, with hyperparameter optimization, most of the predictors however obtained similar accuracies with upper bounds between 70–80%. This upper limit on accuracy for both the linear and nonlinear models leads us to believe that more sophisticated models will not be more successful on the present dataset. Rather, we believe that a more comprehensive and descriptive feature set will be required to obtain more accurate predictors. With a larger feature set, it is conceivable that then more sophisticated models may be necessary for achieving more accurate results.

The engineered features, particularly 3D surface dose, have actual values which the present study only approximated. In the case of 3D surface dose, actual target surface areas can be calculated using treatment planning software, and in the future could be determined this way. Due to limitations of the available datasets, the present study could only approximate the true value of this feature utilizing calculated spherical surface area. A limitation with regards to comparing the present approach to existing models is the fundamental difference in approach towards feature selection. Existing systems, the VRAS, RBAS, and Spetzler-Martin, utilized a small set of pre-treatment features, whereas our current approach attempted to derive the best possible prediction utilizing all available information. In a sense then, the fundamental advantage of a machine learning or data science approach towards making predictive models is a philosophical one with regards to the use of data.

Lastly the predictors for the present study were each developed for a specific time points, two, three, four, six, and eight years. While useful time points clinically, such discrete results are suboptimal, due to a reduction in predictor accuracy the farther time gets from its point of optimization. The current system could stand to be improved upon by creating a system that continuously sweeps over all available time points, or by creating an ensemble model that utilizes a union of the existing discrete predictors and weights predictions inversely to the difference in time from a given point to the optimal.

Variability in patient outcomes after a given therapy is a known part of medical practice. Machine learning presents novel tools to predict outcomes and, in the case of AVM radiosurgery, can provide a powerful tool for treatment planning. This approach towards developing optimal predictors has enabled the present study to create a tool that outperforms existing prognostic systems for predicting AVM outcomes. Point of care prognostics for a specific AVM patient, based upon the outcomes of a particular practice in a given population and harvested automatically from a clinical registry or electronic medical record, may be able to ensure we deliver the right care to the right patient.

## Methods

### Participant selection, definitions, and outcomes assessment

We retrospectively evaluated prospectively maintained, institutional review board (IRB) approved AVM radiosurgery databases from three institutions participating in the International Gamma Knife Consortium (IGKRF). A total of 1,910 patients, comprising 1,010 patients from the University of Virginia (Site #1), 800 patients from the University of Pittsburgh (Site #2), and 100 patients from New York University (Site #3) were de-identified and pooled by an independent third party, and then sent to the institutions of the first and senior authors for analysis. All patients were treated using the Leksell Gamma Knife (Elekta AB), the details of which have been previously reported^38^,^39^,^40^. All patients were required to have sufficient data regarding prior interventions, clinical presentation, AVM characteristics, and post-SRS outcomes, as well as follow-up angiography or MRI in order to be included in the site-specific databases with a minimum follow-up of at least 2 years between the two sites. Radiologic follow-up was obtained by MRI at approximately six to twelve month intervals for the first two years, and then annually thereafter. Obliteration was defined as the absence of flow voids on MRI, or the absence of anomalous arteriovenous shunting on angiography. Angiography to confirm nidal occlusion was performed, when possible, after obliteration was noted on MRI. Latency period hemorrhage was defined as hemorrhage following SRS treatment, regardless of neurological condition. An “unfavorable” outcome was defined as any patient experiencing a post-radiosurgery hemorrhage, or a new, permanent neurological deficit. A “neutral” outcome was defined as a patient at a specified time point with a patent AVM, but not having an unfavorable outcome. Lastly, a “favorable” outcome, the focus of the current study, was defined as any patient achieving obliteration at a specified time point without suffering an unfavorable outcome.

### Initial and engineered features

Features (variables) in the initial database included standard clinical and treatment parameters: gender, age, prior treatments, prior AVM hemorrhage, presence of intranidal or perinidal aneurysms, margin dose, maximal dose, isodose, number of isocenters, duration of follow-up, and clinical symptoms. Angioarchitectural features included location (eloquent vs. non-eloquent and deep vs. superficial), maximum diameter, volume, number of draining veins, and location of draining veins (superficial only vs. any deep component). Age was re-scaled as log age. Location was defined both in terms of critical neurologic function, based on the Spetzler-Martin (SM) grading scale, as well as neuroanatomical region. Regions were defined in terms of the major lobes (frontal, temporal, parietal, occipital), basal ganglia, thalamus, corpus callosum, brainstem, cerebellum, or insula. Feature engineering, a pre-training manipulation of data to boost predictor performance was performed in two steps^41^. Automatic feature engineering was performed by pairwise multiplication and binary re-classification of features, in addition to expert judgement feature engineering performed by a member of the team familiar with both clinical medicine as well as machine learning. Most notably the effort at expert judgement driven feature engineering yielded the *3D surface dose* which, assuming spherical AVM geometry and a roughly homogenous dose distribution, can be approximated as nidus *three dimensional surface area* multiplied by the average radiosurgical *margin dose*.

### Machine learning analysis and statistical comparison

Predictors (predictive models) were constructed to predict favorable outcomes using a machine learning approach. Novel features not present in the initial datasets were constructed as previously discussed. Pre-processing, including normalization and re-classification, was performed for all features in order to assess their utility for inclusion in the final model. All non-binary features were normalized to a mean of zero and unit variance. An iterative process of feature engineering, model generation, and model/feature assessment was performed to identify useful features for prediction. Cases with missing values were excluded from the overall analysis. As part of predictor construction, all predictors were tested both with and without initial optimization (grid search) of their hyper-parameter space by assessing cross-validated accuracy on training subsets of the dataset as described below. Predictors tested in the current study included logistic regression (LR) with L2 regularization, a linear support vector machine (SVM)^42^ with L2 regularization, gradient boosting^43^, Random Forests (RF)^42^, and Extremely randomized trees^44^,^45^,^46^,^47^.

Predictor construction was performed by generating N fixed bootstrap^42^ samples consisting of two-thirds of the dataset, with the remaining one-third reserved for validation (N = 100 samples for the current manuscript). Ten-fold cross validation on each bootstrap training set was performed to optimize predictor hyperparameters (Table S1). Lastly, optimized predictors were trained on the entire bootstrap testing set and tested against the corresponding testing set. The process was performed iteratively across all N bootstrap testing sets and displayed graphically to show the variation within each predictive technique. To quantify each model’s general precision, all N models were averaged to generate a final measure of average accuracy and variance for each predictor. A similar bootstrap sampling process, with omission of a training step, was performed for all standard clinical models as well. Traditional models of AVM outcomes after radiosurgery included in the step were the modified radiosurgery-based AVM score (RBAS)^6^, Virginia Radiosurgery AVM Scale (VRAS)^24^, and Spetzler-Martin grading scale^22^. All predictors and models were evaluated for their predictive accuracy at standard time points of, two, three, four, six, and eight years using area under receiver operating characteristic (AUROC), which is a measure of accuracy that is invariant to class size imbalance. By evaluating survival, a binary outcome, at each specific time point for patients who had achieved an outcome by that given time point, we were able to mimic survival regression across a time series despite utilizing standard machine learning models. As a further testing of predictor accuracy and generalizability, the final predictors were validated against patients from Site #3 that were not involved in initial predictor construction, training, or validation.

In order to utilize the predictive models to also obtain an understanding of the underlying parameter space and how well it characterized the underlying radiobiology, we attempted to ascertain the importance of each feature’s contribution to predictor construction. For each of the finalized N predictors, following training, the features included as part of the predictor were stored in a separate dataset, and tabulated following construction of all N predictors to obtain a frequency analysis of feature usage.

To assess the novel classifiers for potential clinical utility, we identified the group of patients predicted to fail to obliterate by 8 years and analyzed this group’s clinical characteristics. Hemorrhage rates were calculated at various time points and analyzed to see whether classifier predictions could delineate high-risk individuals, as well as whether obvious pre-treatment variables identified this set of high-risk patients. All statistical comparisons were made using an alpha of 0.05. Standard nonparametric statistical testing for univariate data was utilized as appropriate including the Mann-Whitney and Chi-square tests. To identify optimal cutoff points for continuous data to classify specific categories, an automated binning algorithm utilizing the minimal description length principle (MDLP) was employed, as previously described^48^. All data management and analyses were conducted using the open source scikit-learn library in Python.

## Additional Information

**How to cite this article**: Oermann, E. K. *et al*. Using a Machine Learning Approach to Predict Outcomes after Radiosurgery for Cerebral Arteriovenous Malformations. *Sci. Rep.* **6**, 21161; doi: 10.1038/srep21161 (2016).

## Supplementary Material

Supplementary Information

## Figures and Tables

**Table 1:** (A) A summary of existing prognostic systems. Many existing prognostic systems utilize a similar feature set, but differ considerably in their types (continuous vs categorical) and in the predictors constructed with those features in the original manuscripts. **(B)** A summary of the number of patients at each time threshold with patients not having complete data at each threshold being censored.

**Prognostic Scoring System**	**Feature List**	**Feature type**	**Original Model/Predictor**
**A: Summary of existing prognostic systems**			
SM Classification^1^	Maximum nidus diameter	Categorical: 1–3	Quadratic regression (∑ *features*)
	Location (±critical function)	Binary: 0, 1	
	Venous drainage	Binary: 0, 1	
RBAS^1^	Volume	Continuous	Linear regression (∑ *weighted features*)
	Location (deep vs superficial)	Binary: 0, 1	
	Age	Continuous	
VRAS^1^	Volume	Categorical: 0–2	Logistic regression (∑ *features*)
	Location (±critical function)	Binary: 0, 1	
	History of hemorrhage	Binary: 0, 1	
**B: Number of patients at each time threshold**			
**Time Threshold**		**Number of patients**		
Year 2		1674		
Year 3		1586		
Year 4		1442		
Year 5		1386		
Year 6		1340		
Year 7		1308		
Year 8		1291		

^1^SM = Spetzler-Martin; RBAS = modified radiosurgery-based AVM score; VRAS = Virginia radiosurgery AVM scale.

**Table 2:** Frequency of variable inclusion in LR predictor.

**Feature**	**Frequency at t = 2 years**	**Frequency at t = 3 years**	**Frequency at t = 4 years**	**Frequency at t = 6 years**	**Frequency at t = 8 years**	**Avg.**
Maximum diameter (mm)	1	1	1	1	1	1
Prior embolization (yes/no)	0.88	1	1	1	0.99	0.974
Marginal dose (Gy)	0.92	0.97	0.94	0.98	0.92	0.946
Number of isocenters (no.)	0.84	0.81	0.78	0.96	0.95	0.868
Location 8 – brain stem (yes/no)	0.63	0.71	0.9	0.96	0.93	0.826
Associated aneurysm (yes/no)	0.68	0.88	0.84	0.86	0.83	0.818
Location 5 – thalamic (yes/no)	0.77	0.78	0.81	0.88	0.75	0.798
Location 2 – temporal (yes/no)	0.68	0.73	0.91	0.82	0.76	0.78
Surgery (yes/no)	0.81	0.49	0.76	0.86	0.74	0.732
Deep venous drainage (yes/no)	0.69	0.64	0.64	0.85	0.82	0.728
History of Hemorrhage (yes/no)	0.69	0.73	0.62	0.75	0.73	0.704
Sex (male/female)	0.68	0.61	0.69	0.79	0.7	0.694
Age (years)	0.6	0.64	0.6	0.77	0.67	0.656
3D surface dose (Gy × mm^2^)*	0.87	0.71	0.6	0.51	0.59	0.656
Location 9 – cerebellum (yes/no)	0.64	0.76	0.62	0.62	0.59	0.646
Location 1 – frontal (yes/no)	0.5	0.56	0.63	0.71	0.75	0.63
Location 7 – callosal (yes/no)	0.65	0.51	0.61	0.72	0.62	0.622
Location 4 – occipital (yes/no)	0.55	0.42	0.6	0.73	0.8	0.62
Volume (mm^3^)	0.59	0.4	0.57	0.75	0.72	0.606
Location 3 – parietal (yes/no)	0.82	0.58	0.53	0.56	0.47	0.592
Location 6 – BGߤ (yes/no)	0.8	0.51	0.5	0.57	0.57	0.59
Isodose (%)	0.81	0.51	0.49	0.56	0.55	0.584
Max dose (Gy)	0.48	0.4	0.7	0.52	0.59	0.538

The set of utilized features in the logistic regression predictor (LR predictor) and their frequency of model inclusion at different time points. Bolded text indicates the top five utilized features at each time point, with a final column (Avg.) denoting the average rate of inclusion in the predictors across all time points. The majority of features were derived from original patient data, while one feature, *marginal dose* (*Gy*) × *surface area* (*mm*^2^), was consistently selected for inclusion in the learning model and of particular use at predicting early obliteration rates.

^*^The engineered feature selected for inclusion in the model.

^ߤ^basal ganglia.

**Table 3:** A confusion matrix of predictive results from the LR predictor at 4 years for the independent dataset of Site #3.

**Site #3 at 4 years**	**Predicted Unfavorable Outcome**	**Predicted Favorable Outcome**	**Totals**
Unfavorable Outcome	16	10	26
Favorable Outcome	5	29	34
Totals	21	39	60

The LR predictor had a sensitivity of 85%, and a specificity of 62%. These results yield a positive predictive value (PPV) of 74% and a negative predictive value (NPV) of 76%.

**Table 4:** A list of the classifiers employed and their respective hyperparameters which were optimized utilizing a grid-search routine prior to classifier training and testing.

**Classifiers and their respective hyperparameters**
L1-penalized logistic regression
C: [10^4 … 10^2]
L2 -penalized Linear SVM
C: [10^-4 … 10^2]
Random Forest
Max depth : {10, 20, 30}
# trees: {25, 50, 100}
minimum # of split samples: {2, 3, 4, 5}
Extremely Randomized Trees:
Max depth : {10, 20, 30}
# trees: {25, 50, 100}
minimum # of split samples: {2, 3, 4, 5}
Gradient Boosting:
Learning rate: [10^-4 … 1.0]
# boosting stumps: {25, 50, 100}
Depth of each boosting stump: {2, 3, 4, 5}
Subsampling: {0.5, 1.0}
