# Supplementary material for: Using a Machine Learning Approach to Predict Outcomes after Radiosurgery for Cerebral Arteriovenous Malformations
Source: Sci Rep. 2016 Feb 9;6:21161. doi: 10.1038/srep21161 (PMC4746661; doi:10.1038/srep21161)
Supplement: Supplementary Information [file srep21161-s1.pdf]

Using a Machine Learning Approach to Predict Outcomes after Radiosurgery for Cerebral Arteriovenous Malformations

Eric Karl Oermann, M.D.[1†], Alex Rubinsteyn, Ph.D.[2†], Dale Ding, M.D.[3], Justin Mascitelli, M.D.[1], Robert M. Starke, M.D., M.Sc.[3], Joshua B. Bederson, M.D.[1], Hideyuki Kano, M.D., Ph.D.[4], L Dade Lunsford, M.D.[4], Jason P. Sheehan, M.D., Ph.D.[3], Jeffrey Hammerbacher, B.S.[2], Douglas Kondziolka, M.D., M.Sc.[5*]

[1]Department of Neurosurgery, Icahn School of Medicine at Mount Sinai, New York City, NY, USA
[2]Department of Genetics and Genomic Sciences, Icahn School of Medicine at Mount Sinai, New York City, NY, USA
[3]Department of Neurosurgery, University of Virginia Medical Center, Charlottesville, VA, USA
[4]Department of Neurosurgery, University of Pittsburgh Medical Center, Pittsburgh, PA, USA
[5]Department of Neurosurgery, New York University Langone Medical Center, New York City, NY, USA
[†]These authors contributed equally to this work
[*]Corresponding author

Corresponding Author contact information:
Douglas Kondziolka
New York University Langone Medical Center
Department of Neurosurgery
New York, NY 10016
Email Douglas.Kondziolka@nyumc.org

Features	Type	Units
Age	Continuous	years
Associated aneurysm	Categorical - Binary	yes/no
Deep venous drainage	Categorical - Binary	yes/no
History of Hemorrhage	Categorical - Binary	yes/no
Isodose	Continuous	percentage
Location 1 – frontal	Categorical - Binary	yes/no
Location 2 – temporal	Categorical - Binary	yes/no
Location 3 – parietal	Categorical - Binary	yes/no
Location 4 – occipital	Categorical - Binary	yes/no
Location 5 – thalamic	Categorical - Binary	yes/no
Location 6 – basal ganglia	Categorical - Binary	yes/no
Location 7 – callosal	Categorical - Binary	yes/no
Location 8 – brain stem	Categorical - Binary	yes/no
Location 9 – cerebellum	Categorical - Binary	yes/no
Marginal dose	Continuous	Grey (Gy)
3D surface dose	Continuous	Gy x mm^2
Max dose	Continuous	Grey (Gy)
Maximum diameter	Continuous	millimeter (mm)
Number of isocenters	Ordinal	natural numbers
Prior embolization	Categorical - Binary	yes/no
Sex	Categorical - Binary	female/male
Surgery	Categorical - Binary	yes/no
Volume	Continuous	mm^3
